# Isolation and Molecular Characterization of a Model Antagonistic* Pseudomonas aeruginosa* Divulging* In Vitro* Plant Growth Promoting Characteristics

**DOI:** 10.1155/2018/6147380

**Published:** 2018-01-15

**Authors:** Bushra Uzair, Rehana Kausar, Syeda Asma Bano, Sammer Fatima, Malik Badshah, Ume Habiba, Fehmida Fasim

**Affiliations:** ^1^Department of Bioinformatics and Biotechnology, International Islamic University, Islamabad Capital Territory, Islamabad 44000, Pakistan; ^2^Department of Botany, University of Azad Jammu and Kashmir, Muzaffarabad 13100, Pakistan; ^3^Department of Microbiology, University of Haripur, Haripur, Pakistan; ^4^Department of Botany, University of Gujrat, Hafiz Hayat Campus, Gujrat, Pakistan; ^5^Department of Microbiology, Quaid-i-Azam University, Islamabad 45320, Pakistan; ^6^Department of Forestry and Wild Life Management, University of Haripur, Haripur, Pakistan; ^7^Discipline of Biomedical Science, Sydney Medical School, The University of Sydney, Sydney, NSW, Australia

## Abstract

The use of microbial technologies in agriculture is currently expanding quite rapidly with the identification of new bacterial strains, which are more effective in promoting plant growth. In the present study 18 strains of* Pseudomonas* were isolated from soil sample of Balochistan coastline. Among isolated* Pseudomonas* strains four designated as SP19, SP22, PS24, and SP25 exhibited biocontrol activities against phytopathogenic fungi, that is,* Rhizopus microsporus, Fusarium oxysporum, Aspergillus niger, Alternaria alternata,* and* Penicillium digitatum*; PS24 identified as* Pseudomonas aeruginosa* by 16srRNA gene bank accession number EU081518 was selected on the basis of its antifungal activity to explore its potential as plant growth promotion. PS24 showed multiple plant growth promoting attributes such as phosphate solubilization activity, indole acetic acid (IAA), siderophore, and HCN production. In order to determine the basis for antifungal properties, antibiotics were extracted from King B broth of PS24 and analyzed by TLC. Pyrrolnitrin antibiotic was detected in the culture of strain PS24. PS24 exhibited antifungal activities found to be positive for hydrogen cyanide synthase* Hcn BC* gene. Sequencing of gene of* Hcn BC* gene of strain PS24 revealed 99% homology with the* Pseudomonas aeruginosa strain PA01*. The sequence of PS24 had been submitted in gene bank accession number KR605499.* Ps. aeruginosa* PS24 with its multifunctional biocontrol possessions can be used to bioprotect the crop plants from phytopathogens.

## 1. Introduction

*Pseudomonas* are ubiquitous bacteria which are of industrial significance. This is because they exhibit various traits in a wide variety of environments.* Pseudomonas* influences plant and animal pathogens. Use of* Pseudomonas* spp. includes biocontrol and as a plant growth promoter and an efficient bioremediation strain and also for production of array of antimicrobial compounds [1, 2]. Evidence has highlighted many metabolites produced by* Pseudomonas* strains such as antibiotics, enzymes, and volatiles and their key role in controlling an array of fungi causing infections in plants has been used for biofertilizer production and formulation [3, 4]. Toxins liberated from plant pathogenic fungi cause budding infectious diseases leading to epidemics and concerns for food safety [5]. Furthermore, plant pathogenic fungi threaten plant and have been long recognized as a prevalent problem. There are various epidemics reported because of plant disease caused by microorganisms and have threatened food security in history of mankind [6]. Due to the impact of pathogenic fungi on human life, significant research efforts have been directed towards combating fungal infections by identifying novel targets for development of antifungal agents [7, 8]. Many Antimicrobial compounds, surfactants, and plant growth promoters have been reported by potential plant growth promoting strains colonizing plant roots and bacterial secretions regulate the interaction between plants and PGPR leading to distinctive plant growth promotion special effects [9, 10]. Genetically improved strains with greater plant protection traits were also designed [11]. Consortium of* P. fluorescens* with other plant growth promoting beneficial bacteria strains showed promising plant growth promoting traits [12].

In this study, a strain of* Pseudomonas aeruginosa PS24* which is believed to be a plant growth promoting microorganism was obtained from soil samples of Balochistan coastline (Pakistan). Strains were evaluated to see if they had characteristics important for sustainable agriculture. These characteristics include biological control protection, phosphorous solubilization, protease production, HCN, plant growth promoting hormone (indole acetic acid), and siderophore production. HCN gene cluster from* Pseudomonas aeruginosa* PS24 was also amplified and sequenced.

## 2. Material and Methods

### 2.1. Sample Collection and Isolation of* Pseudomonas* Strains

Coastline soils samples were collected from Balochistan coastline of Pakistan. Physicochemical analysis of samples based on soil texture, pH, and temperature was examined. The soil texture was slit clay type, pH in the range of 7.5 to 8.8, and temperature was 25 to 32°C. Microbial strains were isolated by the serial dilution method. One gram of dried soil was weighed and added to 9 ml of double distilled water (dd H_2_O) in a sterile test tube and shaken well using vortex mixer; this stock solution was then diluted serially up to the dilution of 10^−5^ and 0.1 mL of diluted sample was inoculated on surface of selective King's B agar and incubated at 30°C for 2 days [13]. The purified colonies were preserved using standard preservation methods.

### 2.2. Identification of the Selected Bacteria

After isolation of strains on selective King B medium the strains were identified and characterized by morphological, cultural, and biochemical tests using Bergey's manual as a reference [14]. Identification was also confirmed by 16S rRNA gene sequencing by using GAGTTTGATCCTGGCTCAG and AGAAAGGAGGTATCCAGCC forward and reverse primer sequence, respectively.

### 2.3. Production of Antifungal Compounds by* Pseudomonas* Strains

In this study all together 18 strains of* Pseudomonas* were examined for exploring antifungal activity of strains by dual culture assay using method described by [15].

### 2.4. Antimicrobial Metabolites Produced by PS24

Ability of PS24 to produce siderophore was examined using method described by Alexander and Zuberer 1991 [16]. Using Bakker and Schippers 1987 [17] method HCN production was determined.

### 2.5. Determination of Phosphorous Solubilization and Protease Production Activity

The phosphate solubilization ability of* Pseudomonas* PS24 strain was determined by using, Mehta and Nautiyal, protocol [18]. A single colony was streaked onto the tris minimal medium supplemented with 0.8 mM calcium triphosphate and 0.5 mM glucose. After 3 days of incubation at 30°C, a clear zone around the colony was observed and measured. Protease production was done on skim milk agar plates containing 2.5% skim milk.

### 2.6. Estimation of Inorganic Phosphate by HPLC

At different intervals of time release of free inorganic phosphate in tris minimal liquid medium with the addition of 5 mM calcium triphosphate by PS24 was determined by HPLC. Aseptically drawn 10 ml aliquot from each flask was taken and centrifuged at 5000*g* for 15 min. Pellet was discarded and supernatant was assessed for phosphate content using column (AS11-HC/AG11-HC). The phosphate content was quantitatively analyzed by comparing the retention times and peak areas of chromatograms with standards.

### 2.7. Screening for Indole Acetic Acid (IAA) Production

Method of Ehmann, 1977, was used to screen IAA production using Salkowski's reagent [19]. The strain* Pseudomonas PS24* was grown in tryptone soy broth and incubated for 4 days. The broth was centrifuged and supernatant was mixed with 2 ml of Salkowski's reagent and kept in the dark. Development of pink color is indicative of indole acetic acid (IAA) production [19].

### 2.8. Analysis of Antibacterial Compounds from PS24

From PS24 grown on King B broth antimicrobial compounds were extracted using combination of extracting solvents, ethyl acetate, and 80% acetone in equal volume. TLC of the prepared crude extract was performed with precoated silica gel G25-UV254 plates and was observed under UV light (254 nm). The distinct separated fractions were recovered in DMSO and tested for antifungal activity.

### 2.9. Exploring Antifungal Compound Producing Genes of* Pseudomonas* and* Its Sequencing*

Selected strain producing highest zone of inhibition was subjected to the detection of Hcn BC gene using Primer Aca and Acb of Ramette et al., 2003 [20]. The sequence of primers for PCR amplification used was Aca ACTGCCAGGGGCGGATGTGC and Acb ACGATGTGCTCGGCGTAC. Sequencing of Hydrogen Cyanide Synthase Gene of* Pseudomonas* was also done. Sequencing was run using ABI3130 Genetic Analyzer.

## 3. Results

### 3.1. Isolation and Identification of Antagonistic Bacteria Strains

All 18 strains of* Pseudomonas* recovered from soil samples of Balochistan coastline were Gram-negative and positive for citrate, oxidase, catalase-, indole, and produced siderophores and inert reaction was observed on triple sugar iron agar slants. The strain was identified as* Pseudomonas aeruginosa* accession number EU081518. These strains were screened for antifungal activity to inhibit growth of fungal strains. Four strains showed effectual antagonistic activity towards* Rhizopus microsporus*,* Fusarium oxysporum, Aspergillus niger, Alternaria alternata,* and* Penicillium digitatum*. Among them, the bacterial strain PS24 was the leading one as antagonist strain against fungal pathogens. Therefore PS24 strain was used for further experiments in search of potential strain that can be employed as biofertilizer strain. The colony of strain of PS24 was orangish yellow pigmented, shiny, and exhibiting an irregular margin. Identification characteristics are presented in (Table 1).

### 3.2. Assessment of Antifungal Activity

**Figure 1:**
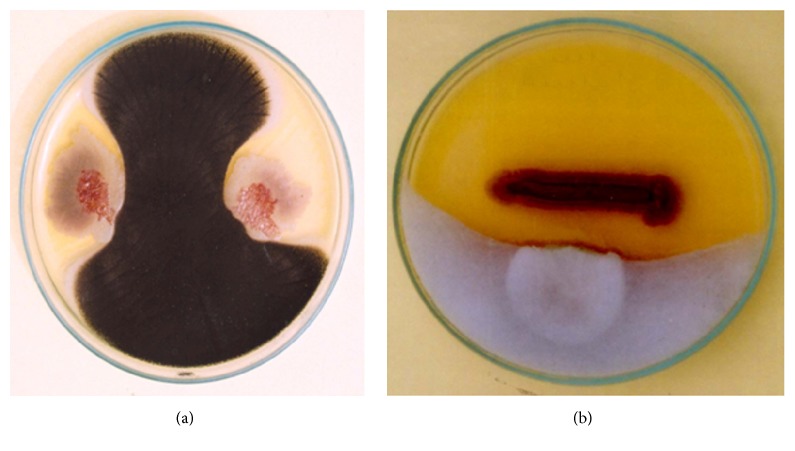
Screening of* Pseudomonas* strains PS24 for antifungal activity against plant pathogens, (a) inhibition of* Alternaria alternata;* (b) inhibition of* Rhizopus microsporus*.

The cross-streak method and dual culture assay of the culture/culture filtrate of strain PS24 showed antifungal nature by both tested methods for evaluation of antifungal potential. Antifungal activity was observed with* Fusarium oxysporum, Aspergillus niger, Alternaria alternata, *and* Penicillium digitatum* (Figure 1; Table 2).

### 3.3. Screening for Plant Growth Promoting Characteristics of PS24

**Figure 2:**
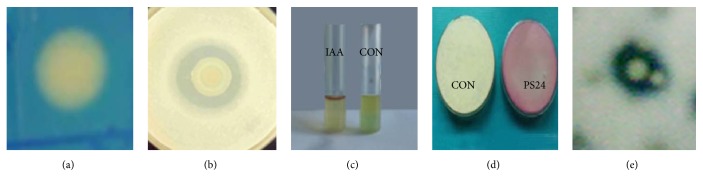
Plant Growth Promoting Characteristics of strain PS24. (a) Siderophore production. (b) Phosphate solubilization. (c) Indole acetic acid IAA production. (d) HCN production. (e) protease production.

Both the phosphate solubilizing strains showed manifestation of a reddish-brown zone around the colony on CAS agar plates which confirmed the siderophore production (Figure 2). This strain also displayed production of indole acetic acid (Figure 2).

### 3.4. Estimation of Inorganic Phosphate by HPLC

**Figure 3:**
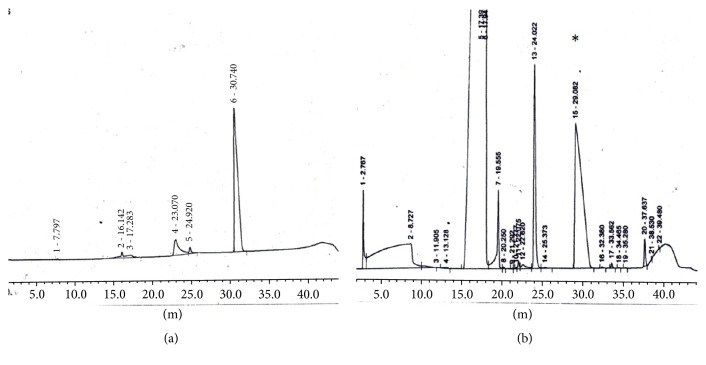
HPLC chromatogram showing free phosphate release. (a) Standard of phosphate (5 mM), (b) phosphate release by PS24 as a result of solubilization of calcium triphosphate in tris minimal medium supplemented with 5 mM calcium triphosphate.* Key*. The peak that elutes at 30 min (indicated with an asterisk) represents phosphate. (a) Standard of phosphate. (b) Phosphate release at 10th day of incubation.

The presence of soluble phosphate in the medium after different intervals of time (0 day, to 20 days) was confirmed by HPLC. Results are shown in (Figure 3). PS24 solubilized calcium triphosphate in liquid medium with connected release of free phosphate in the medium HPLC analysis showed that the quantity of phosphate in the medium demonstrated a steady boost and attained the highest level after 10 days of incubation.

### 3.5. Chromatographic Identification of the Antibiotic Produced by Strain PS24

**Figure 4:**
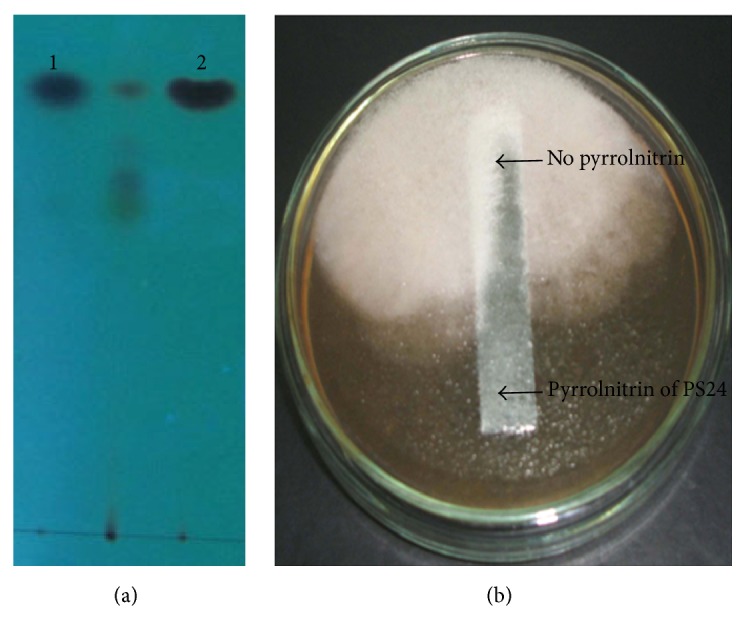
(a) TLC showing compounds produced by* Pseudomonas* strains Lane 1: Pf-5 reference strain; Lane 2: PS24. (b) Antifungal activity of pyrrolnitrin of PS24 separated on TLC.

Antimicrobial compounds extracted out from strain PS24 and reference strain Pf-5 grown in King B were spotted on TLC reverse phase silica gel plates that illustrated a pyrrolnitrin band as produced by reference strain Pf-5 (Figure 4). Separated pyrrolnitrin compound on TLC plate showed antifungal activity (Figure 4).

### 3.6. Screening for the Antifungal Compound Producing by PCR and Sequencing of Hydrogen Cyanide Synthase Gene

**Figure 5:**
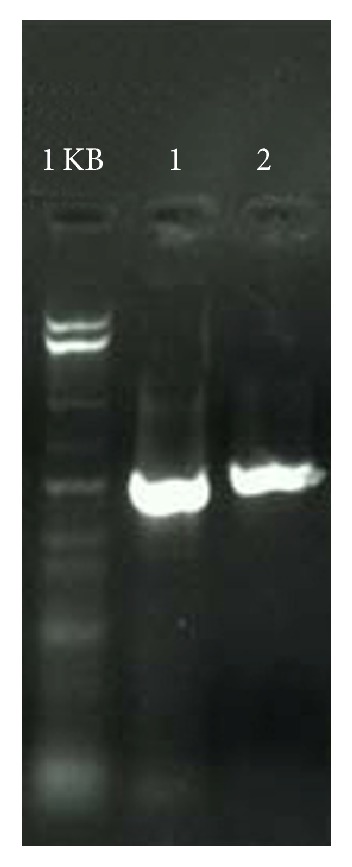
Hcn gene Amplification by PCR, strains positive for Hcn gene showed band size of approx. 586 bp. Lane 1 contains PCR product of reference strain Pf5 and lane 2 contains PCR product of PS24.

Four potential biocontrol strains showed clear bands on agarose gel for* Hcn BC* gene with known primers Aca and Acb and the band size was approx. 586 bp (Figure 5). Sequencing of Hydrogen Cyanide Synthase Gene showed 99% homology with the* Pseudomonas aeruginosa* strain PA 01. The sequence of PS24 had been submitted in gene bank and the accession number of this sequence is KR605499.

## 4. Discussion

Pakistan's 1050 km coastline stretches along the Sindh and Balochistan Province. The greater stretch being in Balochistan of 800 km and the remainder 250 km stretch along Sindh, Pakistan, receives less than 250 mm of rainfall annually which makes the climate semiarid and arid. The coastline is very diverse with regard to marine flora and fauna. Commercially important species can be found intertidal, near the shore and on off shore areas too [21]. Evolution of a variety of highly adapted living organisms is due to the temporal and climatic selection process. These living organisms are a wealthy source of microbial diversity [22]. Unfortunately, very few studies reflect on the diversity of microorganisms found in Balochistan coastline. The present study is based on exploration of potential biofertilizer strains from soil samples of Balochistan coastline of Pakistan. Bacterial plant growth promotion is a complex, well-known and established phenomena. A variety of phenotypic and genotypic characteristics are involved in influencing plant growth in a vast array of agricultural environments as indicated by plant growth promoting bacterial strains [23, 24]. In the present study,* Pseudomonas* strains were screened for their potential to be used for sustainable agriculture as a possible biocontrol and plant growth promoting agent. Among the isolated strains* Pseudomonas* strain PS24 identified as* Pseudomonas aeruginosa* was studied for holding a unique plant growth promoting activity. This was based on its biocontrol property and phosphorous solubilizing activity. This property has been appraised and key information proposes insight into this organism's alternative of biocontrol capabilities.* Pseudomonas* spp. are distinguished by their production of colorful secondary metabolites called phenazines [25] and strain PS24 produced yellowish orange pigments in King B agar medium. Antimicrobial compounds were extracted from PS24 grown on King B medium and separated on TLC plates. The distinct separated fractions revealed antifungal potential as shown in Figure 4. In this study, we have obtained efficient detection of pyrrolnitrin on TLC when compared with other known strains. Another significant attribute could be better availability of phosphorous because of P-solubilization by the strain PS24 both on solid and liquid medium as confirmed by plate assay and HPLC analysis in liquid tris minimal medium supplemented with calcium triphosphate. It is well established that enhanced phosphorous nutrition powers general plant growth and root maturity of various economically important plants. Siderophore for iron provision of plants grown under iron scarce conditions and HCN production is measured valuable from the biocontrol viewpoint. Many studies have shown and have been carried out to highlight the nature and properties of specific microbes which exhibit plant growth promoting properties. Along with chemical fertilizers it is important to seek region-specific strains of microorganisms which can be used in plant growth promotion [26].

Paulsen et al., 2005, [27] showed the complete genome sequence of the* Pseudomonas* fluorescens Pf-5 (a plant commensal). In this study the presence of* Hcn* BC gene was also explored. Furthermore, sequencing of* Hcn BC* gene was done. Rijavec and Lapanje, 2016, [28] reported hydrogen cyanide composite is not vital or necessary for progress, energy stowing, or primary metabolism in* P. aeruginosa* but it can offer some environmental advantages to the bacterium by regulating availability of phosphate. De Souza et al., 2003, [29] reported conservation of the response regulator gene* gacA* in* Pseudomonas* species. Whole genome sequences are now available for two biocontrol strains belonging to the* P. fluorescens* lineage (strain SBW25) or a closely related species (strain Pf-5), as well as non-biocontrol* P. fluorescens* Pf0-1. Comparative genomics [9], gene array-based expression studies [30], and integrated, in situ molecular analyses of microbe-host interactions [4] have started to provide advanced knowledge on plant protection properties and rhizosphere competence of these biocontrol agents. The present study is an attempt to untangle secondary metabolism in* Ps. aeruginosa* strain PS24 that may have a direct role in its ability to suppress fungal pathogens.

## 5. Conclusion

Antagonistic* Pseudomonas aeruginosa* PS24 isolated from soil sample of Balochistan coastline of Pakistan have shown promising biocontrol abilities, which are closely linked with the production of antifungal compound Pyrrolnitrin This is one of the few reports dealing with isolation and characterization of* Pseudomonas aeruginosa* strain from soil sample of Balochistan coastline with biocontrol activity against the common soil-borne phytopathogenic fungi in this context; it is proposed that* Pseudomonas aeruginosa* PS24 can be deployed as an inoculant to attain the desired plant growth promoting action in agricultural environment. Hence it is important to search for region-specific microbial strains which can be used as a potential plant growth promoter to achieve desired product. In this study, we reported* Pseudomonas aeruginosa* strain with the production of HCN, Pyrrolnitrin, IAA, P-solubilization, and siderophore production as a potential plant growth promoting strain and can be used as bioinoculants in agricultural environments. Thus, the joint developments in environmental biotechnology will support us to comprehend the establishment of* Pseudomonas* strains as model biocontrol agents, and their right and on time use in rhizosphere and understanding of their behavior and performance in natural soil system for the growth promotion of economically important plants might help us overcome existing tailback curbing their commercial use.

## Figures and Tables

**Table 1:** Biochemical characteristics and estimation of various biological traits of PS24.

Test	Trait
Auxin production	Positive
Siderophore	Positive
Phosphorous solubilization	Positive
HCN production	Positive

*Antifungal activity*	

*Rhizopus microspores*	++++
*Fusarium oxysporum,*	+++
*Aspergillus niger*	++
*Alternaria alternata*	++
*Penicillium digitatum*	+

**Table 2:** *In vitro* antifungal activity of PS24.

Test fungi	Size of zone of inhibition (mm)	Size of zone of inhibition (mm)
	*Culture filtrate of PS24*	*Crude extract of PS24*
*Candida albicans*	-	-
*Candida tropicalis*	-	-
*Rhizopus microsporus*	15.5	16.0
*Fusarium oxysporum,*	14.8	15.0
*Aspergillus niger*	16.6	18.4
*Alternaria alternata*	14.5	16.5
